# Immature rat seminal vesicles show histomorphological and ultrastructural alterations following treatment with kisspeptin-10

**DOI:** 10.1186/1477-7827-10-18

**Published:** 2012-03-10

**Authors:** Faiqah Ramzan, Irfan Zia Qureshi, Muhammad Ramzan, Muhammad Haris Ramzan, Faiza Ramzan

**Affiliations:** 1 Gomal Centre of Biochemistry and Biotechnology, Gomal University, Dera Ismail Khan 29050, Pakistan; 2 Laboratory of Animal Physiology, Department of Animal Sciences, Faculty of Biological Sciences, Quaid-i-Azam University, 45320 Islamabad, Pakistan; 3 Department of Biochemistry, Peshawar Medical College, Peshawar, Pakistan; 4 Institute of Basic Medical Sciences, Khyber Medical University, Peshawar, Pakistan; 5 Laboratory of Microbiology, Department of Microbiology, Faculty of Biological Sciences, Quaid-i-Azam University, 45320 Islamabad, Pakistan

## Abstract

**Background:**

Degenerative effects of critical regulators of reproduction, the kisspeptin peptides, on cellular aspects of sexually immature male gonads are known but similar information on accessory sex glands remain elusive.

**Methods:**

Prepubertal laboratory rats were injected kisspeptin-10 at three different dosage concentrations (10 pg, 1 ng and 1 microgram) for a period of continuous 12 days at the rate of two doses per day. Control rats were maintained in parallel. The day following the end of the experimental period, seminal vesicles were removed and processed for light and electron microscopic examination using the standard methods. DNA damage was estimated by DNA ladder assay and DNA fragmentation assay.

**Results:**

The results demonstrated cellular degeneration. Epithelial cell height of seminal vesicles decreased significantly at all doses (*P *< 0.05). Marked decrease in epithelial folds was readily noticeable, while the lumen was dilated. Ultrastructural changes were characterized by dilatation of endoplasmic reticulum and Golgi complex, heterochromatization of nuclei, invagination of nuclear membranes and a decreased number of secretory granules. Percent DNA damage to the seminal vesicle was 19.54 +/- 1.98, 38.06 +/- 2.09 and 58.18 +/- 2.59 at 10 pg, 1 ng and 1 microgram doses respectively.

**Conclusion:**

The study reveals that continuous administration of kisspeptin does not lead to an early maturation but instead severe degeneration of sexually immature seminal vesicles.

## Background

In 1999, Lee and colleagues discovered in the rat a novel G protein-coupled receptor, the GPR54. The GPR54 gene encodes a G protein-coupled receptor [1]. It was later shown to mediate the actions of a unique family of KiSS-1 derived endogenous ligands known as kisspeptins. The KiSS-1 gene encodes a 145-amino acid peptide that is cleaved into an amidated C-terminal 54 amino acid product, kisspeptin or metastin. Shorter fragments of kisspeptin-54, the kisspeptin-14, kisspeptin-13, and kisspeptin-10, also bind to GPR54. Kisspeptin-54 was originally identified as metastasis suppresser peptide from malignant melanoma cells that had been suppressed for metastatic potential by the introduction of human chromosome 6, hence named metastin [2-4]. In 2003 two independent groups showed that dysfunctional or deletional mutations in the gene encoding the G protein-coupled receptor, GPR54, cause hypogonadotropic hypogonadism, a condition characterized by absent or delayed pubertal development in humans and mice [5,6].

Kisspeptin secreting neurons are found in the arcuate nucleus (Arc), the periventricular nucleus (PeN), and the anteroventral periventricular nucleus (AVPV) in mice [7-9]. Expression of both KiSS-1 and GPR54 mRNA is regulated developmentally as well as hormonally, with a sharp increase at prepubertal age in both male and female rats, changes throughout the estrous cycle in adult females, and increases after gonadectomy that is prevented by sex steroid replacement in both males and females [10,11]. Kisspeptin potently release LH in mice and rats, in both males and females, and in prepubertal, pubertal, and adult rats, as well as in juvenile agonadal male monkeys [7,10,12,13].

When kisspeptin acts at the level of the hypothalamus to increase GnRH secretion, it produces an increase in LH release from the pituitary. However, some studies suggest that kisspeptin may also act at the level of the pituitary to evoke LH secretion through a direct action on the gonadotropes [14]. The presence of a functional kisspeptin receptor in the pituitary, combined with the finding that kisspeptin is released in ovine hypophyseal portal blood, suggests kisspeptin action at the level of the pituitary to modulate gonadotropin secretion [15].

After an initial stimulation, a continuous (chronic) exposure of the pituitary to GnRH (or agonists) eventually causes suppression of gonadotropin secretion [16] through down-regulation and desensitization of the GnRH receptors [17-20]. Continuous delivery of exogenous kisspeptin appears to desensitize Kiss1r, resulting in decreased LH secretion in agonadal juvenile and adult male monkeys and testicular degeneration in adult male rats [21-23]. In contrast, repeated peripheral injections of kisspeptin elicit unrestrained LH pulses in male rats and monkeys [24,25], implying that the efficacy of kisspeptin to drive LH secretion depends on its pulsatile nature.

Besides testes, the seminal vesicles are important androgen dependent accessory sex glands [26]. The seminal vesicles are elongated saccular organ with numerous lateral outpocketings from an irregularly branched lumen. They arise as evaginations of the ductus deferens. The wall consists of an external connective tissue layer rich in elastic fibers, a middle layer of smooth muscle and an epithelium resting upon a layer of loose connective tissue. The mucosa forms an intricate system of thin, primary folds, which branch into secondary and tertiary folds. These project far into the lumen and anastomose frequently. In this way numerous cavities in different sizes are formed and separated by thin branching partitions. All of these cavities open into central cavity, but in sections many of them may seem to be isolated [27]. The epithelium is pseudostratified and consists of rounded basal cells lodged between larger cuboidal or low columnar cells. The epithelial cells contain numerous secretion granules. The secretion of seminal vesicle is a slightly yellowish, viscid liquid. Seminal vesicles mainly secrete prostaglandins and fructose. In sections it appears as coagulated, deeply staining masses in the lumen. The muscular wall of the seminal vesicles is provided with a plexus of nerve fibres and contains small sympathetic ganglia [27].

The seminal vesicles fluid has 5 alpha-reductase activity, which converts testosterone to dihydrotestosterone, the active hormone. Seminal vesicles contain LH/hCG receptors making them a potential target of direct regulation by LH [28]. Seminal vesicles' growth and sex differentiation are both androgens dependent [29,30].

The present study was undertaken to see if kisspeptin can stimulate an early maturation of seminal vesicles. As pharmacological effects of kisspeptin on seminal vesicle remain to be elucidated, the objective of the present study was to investigate in sexually immature male rats, the effect of a range of doses of kisspeptin on the histomorphology and ultrastructure of seminal vesicles, following the intraperitoneal administration of mammalian kisspeptin, kisspeptin-10 (KP-10). We used kisspeptin-10, a 10-residue peptide, because it is the shortest and most active *KISS1 *gene product [2]. It has been suggested that kisspeptin-54 is unstable and may be proteolytically cleaved into the shorter products [2].

## Methods

### Animals and housing

Five weeks old prepubertal male Sprague-Dawley rats having an average body weight of 100 g were procured from the National Institute of Health, Islamabad, Pakistan and maintained in the animal house facility of Quaid-i-Azam University, Islamabad. Five rats were housed per cage (15^// ^× 11^// ^× 9^//^, steel mesh cages) to minimize stress due to crowding. They were provided 12 h Light:Dark photoperiod using the automatic timers, while the room temperature was thermostatically controlled at 25 ± 2°C. Standard rat diet and water were provided *ad libitum*. Animal handling and sacrifice were done according to the guidelines provided by the Ethics Committe of the Department of Animal Sciences, Faculty of Biological Sciences, on care and use of animals for scientific research. European Union guidelines for humane use of animals for experimental purposes were also followed. All chemicals were of analytical grade and were imported from Sigma Chemical Co. (St. Louis Missouri, USA) or BDH (Merck, Germany).

### Experiment: administration of variable kisspeptin doses to prepubertal rats

#### Dose and treatment

Kisspeptin (45-54), obtained as lyophilized powder from Calbiochem (EMD Biosciences, Inc. La Jolla, CA) was dissolved in 1 ml dimethylsulphoxide (DMSO) to give a stock solution of 1 mg ml^-1 ^that was further diluted with distilled water and administered intraperitonealy (i. p.). Animals were randomly assigned to four groups (n = 10 in each). Group I rats constituted control and received 0.9% w/v physiological saline, (DMSO was added to saline at the same concentration as it was added to kisspeptin stock, and was further diluted to concentration equivalent to the experimental doses), group-II rats received 10 pg (15 pmol), group-III rats received 1 ng (1.5 nmol) and group IV received 1 μg (1.5 μmol) kisspeptin as twice daily dose, after every 12 hr for 12 consecutive days. We administered kisspeptin twice daily taking into consideration the plasma half life of kisspeptin. The plasma half-life of kisspeptin-54 was calculated to be 27.6 +/- 1.1 min. The mean metabolic clearance rate was 3.2 +/- 0.2 ml/kg × min, and the volume of distribution was 128.9 +/- 12.5 ml/kg [31]. Kisspeptin doses were selected as previously described Tovar et al. [24]. Toward the end of experiment, 3 hrs after the last dose of the peptide, animals were anesthetized with sodium pentobarbital (60 mgkg^-1 ^_b.w_. i.p). Seminal vesicles were dissected out. The excised tissues were weighed and processed for light and electron microscopy. For light microscopy, seminal vesicles were fixed in freshly prepared chilled 4% paraformaldehyde (pH 7.2); for electron microscopy, these tissues were fixed in 5% glutaraldehyde prepared in 0.2 M pipes buffer. Tissues were also frozen in liquid nitrogen and stored at -80 for estimation of DNA damage.

#### Light microscopy

For histological preparations, tissue samples were fixed and dehydrated in ascending grades of ethanol, cleared in xylene, and embedded in paraffin wax. Thick sections (5 μm) were cut on a microtome (Shandon finesse 325, Italy). Sections were stained with conventional Harris's Hematoxylin and eosin, and mounted in DPX (BDH, Germany) mountant medium.

#### Electron microscopy

Tissues fixed in 5% glutaraldehyde were post fixed in 1% osmium tetraoxide and then in 5% uranyl acetate solution, washed and dehydrated in ascending grades of acetone. They were infiltrated with a mixture of Spur embedding media. The ratio of resin to acetone was 1:3 for 18 hrs followed by 1:1 ratio and then 3:1 for another 18 hrs each. A 100% resin mixture was added to the samples and vacuum infiltration was carried overnight. The samples were then oriented in moulds and resin cured at 70°C for 48 hrs. The polymerized resin blocks were trimmed with fine scalpel blade and glass knife. Ultrathin serial sections of 120 nm were cut with RMC MT 7000 ultramicrotome and placed on 200 mesh nickel grid. Sections were stained with 5% uranyl acetate for 30 min and then washed twice with distilled water. They were then stained with lead citrate for 15 min in NaOH chamber. Sections were examined with a Transmission Electron Microscope (JEOL JEM1010, Japan) operating at 80 kv, at the National Institute for Biotechnology and Genetic Engineering (NIBGE) Faisalabad.

#### Tissue analysis

Tissue sections for light microscopy were observed and photographed using a Nikon optiphot BH 2 research microscope (Japan) with attached camera. Photograph panels were prepared using the Adobe Photoshop (version 7). Epithelial height of cells was measured with an ocular micrometer calibrated with a stage micrometer. Due to the variability in the epithelial cell shape, the epithelial cell height was measured at a place where the epithelial cells stood erect on the basement membrane.

### DNA extraction

DNA was isolated according to the method of Gilbert et al. [32]. At least 30 mg of seminal vesicle tissues were washed twice with 1 ml TE buffer (20 ml of 1 M Tris pH 8.0, 20 ml of 0.5 M EDTA, Sterile distilled water 100 ml). Tissues were ground and 300 μl lysis buffer (1 M Tris HCl pH 8.0, 25 mM EDTA, 100 mM NaCl, 0.1 mg/ml protein kinase) and 240 μl 10% SDS were added, vortexed gently and incubated overnight at 45°C in a water bath. 200 μl of phenol was added, shaken vigorously for 5 min, and centrifuged at 3000 rpm for 5 min. Supernatant was then pipetted out into a new tube, 200 μl phenol and 200 μl chloroform/isoamyl alcohol (24:1) were then added, vortexed and centrifuged at 3000 rpm for 5 min. Supernatant were again pipetted out into a new tube and 25 μl of 3 M sodium acetate (pH 5.2) and 5 ml of ice cold 100% ethanol were added and tubes were kept for overnight at -20°C and then centrifuged for 30 min. The solution was pipetted out gently to avoid disturbance to the DNA. DNA was washed in 70% ethanol and dried in oven at 30°C. 20-50 μl TE buffer (10 mM Tris pH 8.0, 1 mM EDTA) and 2 μl RNase were added. Electrophoresis was performed using 1% agarose resolving gel in 1 × TBE (Tris 89 mM, borate 89 mM and EDTA 2 mM) containing 1 μg ml^-1 ^ethidium bromide (10 mg ml^-1 ^distilled water). Electrophoresis was performed for 45 min at 100 V. The gel was viewed under gel doc system (BIORAD; Germany) and photographed.

### DNA ladder assay

Electrophoresis was performed as above for 45 min at 100 V. 5 μg total DNA per well was loaded. 100 bp DNA ladder was loaded to identify the size of the DNA fragment. The gel was viewed as above.

### Quantitation of DNA fragmentation

DNA quantification was done according to the method of Boraschi and Maurizi [33]. Seminal vesicles tissues (30 mg each) were ground in 1 ml TTE solution (pH 7.4 with 0.2% Triton X- 100 (4°C), 5 mM Tris-HCl, 20 mM EDTA) to make cell suspension and kept overnight at 37°C. Cells were centrifuged at 2000 rpm for 10 min. Supernatants were transfered carefully into new tubes labeled "S". To the pellet in tubes "B" 1.0 ml TTE solution was added and thoroughly vortexed. To separate fragmented DNA from intact chromatin, tubes B were centrifuged at 20,000 × *g *for 10 min at 4°C. Supernatants were transferred carefully to new tubes labeled "T". To the pellet in tubes B, 1.0 ml TTE solution was added. 1.0 ml of 25% TCA was added to tubes T, B and S and vortexed vigorously. Precipitation was allowed to proceed overnight at 4°C. After incubation, precipitated DNA was recovered by pelleting for 10 min at 20,000 × *g *at 4°C. Supernatants were aspirated and DNA was hydrolyzed by adding 160 μl of 5% TCA to each pellet and heating 15 min at 90°C in a heating block. A blank with 160 μl of 5% TCA alone was prepared. To each tube 320 μl of freshly prepared Diphenyleamine solution was added, vortexed and allowed to develop color for about 4 h at 37°C or overnight at room temperature. Optical density at 620 nm was read in a spectrophotometer. The percentage of fragmented DNA was calculated using the formula:

$$\%\;FragmentedDNA=\frac{T\times 100}{T+B}$$

### Statistical analysis

Results were expressed as mean ± SE. The results obtained were analyzed and compared by one way ANOVA followed by post hoc Tukey's adjustment using the Statistical Package for Social Sciences (SPSS, version 16, Inc, Chicago, Illinois, USA). *P *< 0.05 was considered to be statistically significant. Data have presented as mean and standard error of mean (SEM).

## Results

### Effect on seminal vesicles weight

Seminal vesicles weight decreased significantly (*P *< 0.01) at 1 μg dose after 12 days treatment, while at 10 pg and 1 ng doses the decrease was non-significant (Table 1).

**Table 1:** Effect of kisspeptin on seminal vesicles weight (mg) in different experimental groups of prepubertal male rats.

	Control	Kisspeptin (10 pg)	Kisspeptin (1 ng)	Kisspeptin (1 μg)
Seminal vesicle	159.20 ±	128.60 ±	121.00 ±	106.81 ±
weight (mg)	14.74	8.71	7.93	5.20**

(Sample size = 10, mean ± SEM).

Difference from Control = **P < 0.01 compared with control

### Epithelial height

**Figure 1:**
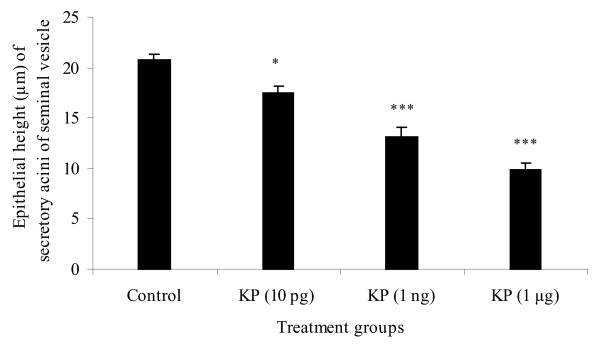
**Epithelial height of secretory acini of seminal vesicle of prepubertal male rats following treatment with 10 pg, 1 ng and 1 μg kisspeptin (KP) doses**. Epithelial height decreased dose dependently. At higher dose the decrease in mean epithelial height of secretory acini of seminal vesicles was more pronounced. Values are expressed as mean ± SE. *P < 0.05, ***P < 0.01 compared to control.

Mean epithelial height of secretory acini of seminal vesicles decreased significantly with kisspeptin treatment. It decreased significantly at 10 pg dose (*P *< 0.05) and at I ng and 1 μg kisspeptin dose the decrease was even greater (*P *< 0.001) (Figure 1).

### Histomorphology of seminal vesicles

**Figure 2:**
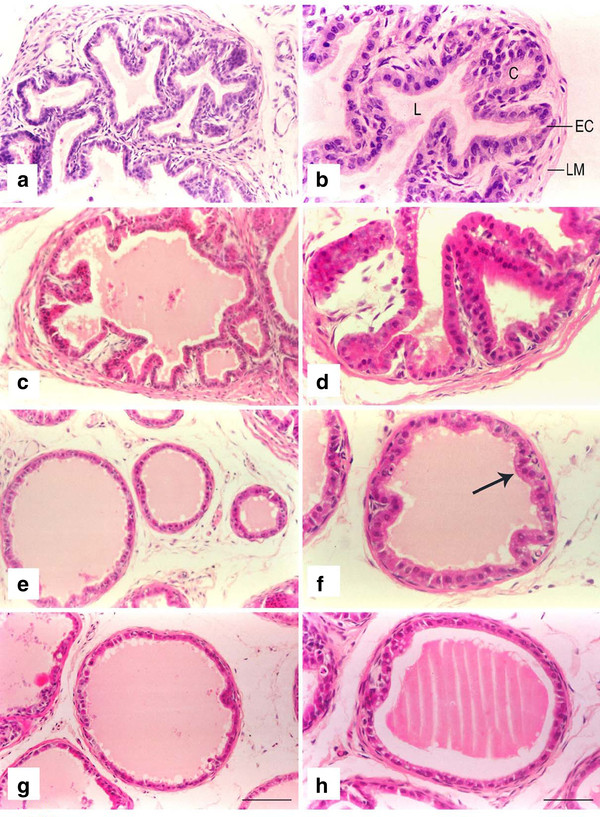
**Photomicrographs of seminal vesicle of control and kisspeptin treated prepubertal rats**. **a-b**: Control seminal vesicle showing complex glandular structure. The lumen (L) is highly irregular having crypts (C). The epithelial cells (EC) are columnar or pseudostratified columnar resting on lamina muscularis (LM)**. c-d**: Seminal vesicle of rats treated with 10 pg kisspeptin. The structure still represents complex foldings, is irregular with dilated lumen as compared to the control. The epithelium is still columnar**. e-f**: Seminal vesicle of rats treated with 1 ng kisspeptin. Seminal vesicles show loss of intricate foldings (arrow) while the lumen is well dilated. The glandular epithelium is not well developed and height of columnar cells appears greatly reduced. **g-h**: At 1 μg kisspeptin dose the lumen has significantly dilated, while the branching of the mucosa of seminal vesicles was distinctly reduced. Compared to low doses of kisspeptin, the epithelial cells are no longer columnar. They were either cuboidal or squamous shape. Scale bar left panel = 50 μm, right panel = 20 μm.

The seminal vesicle of the control group was complex and glandular, and the lumen was highly irregular. The mucosa of the seminal vesicle exhibited thin, branched and anatomizing folds. The epithelia had a variable appearance, it was columnar or pseudostratified columnar. The pseudostratified epithelium of secretory acinus rested on thin basement membrane highly folded to form irregular crypts and peaks. Two types of epithelial cells were recognizable, the principal and basal cells. Principal cells were tall, columnar containing round or oval nuclei and have faintly eosinophilic cytoplasm. Basal cells were present on the basal lamina and were sandwiched between principal cells and had a round or oval nuclei (Figure 2a-b).

Seminal vesicle of rats treated with 10 pg kisspeptin had complex and irregular structure with some alterations as compared to control. The epithelium was still columnar with an irregular lumen (Figure 2c-d).

In the 1 ng treatment group, the seminal vesicle tissue became simple without intricate folds, with the lumen was now dilatated. The glandular epithelium was degenerated and the height of columnar cells was reduced (Figure 2e-f).

Seminal vesicles treated with 1 μg kisspeptin showed markedly dilated lumen and the branching of the mucosa of seminal vesicles was also reduced highly compared to those treated with low doses of kisspeptin. The mucosal epithelia now contained cuboidal or squamous cells (Figure 2g-h).

### Electron microscopic examination

Secretory epithelium of seminal vesicle had two main types of cells, the principal and basal cells. Principal cells were rich in cell organelles. They had prominent nuclei of columnar, oval, round or irregular shape. Nuclei were situated at different heights in the epithelium. Nuclei contained prominent nucleoli and sometimes nuclear bodies of moderate electron density. Chromatin was clumped towards nuclear periphery. Principal cells were rich in mitochondria especially in the perinuclear area. They had a spherical or ovoid shape and were of variable size. The mitochondrial matrix was of low electron density and showed granulated appearance. Mitochondrial granules were seldom seen and were inconspicuous. Endoplasmic reticulum was abundant in these cells. Golgi apparatus was present in the supranuclear region and occasionally seen in other regions. They formed structures consisting of parallel laminae, small vesicles and vacuoles of various sizes. The vacuoles contained dense secretory granules which rarely fill them completely. Secretory granules were abundant in the apical portion of principal cells mainly among Golgi apparatus. There were present some lipid droplets and vacuoles. Ribosomes were present. Twisted, irregular finger shaped microvilli were present at luminal border of principal cells. Microvilli are projections of cytoplasm.

**Figure 3:**
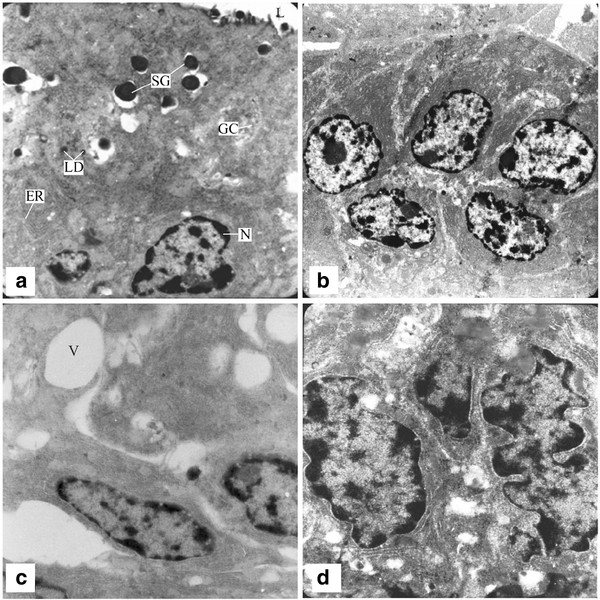
**Electron micrographs of seminal vesicle of control and kisspeptin treated rats**. **a**: A principal cell containing secretory granules, Golgi complex and endoplasmic reticulum. There were present some lipid droplets (LD), large dense core secretory granules (SG) and ribosomes (R). Twisted, irregular finger shaped microvilli were present at luminal border of principal cells. ×12,000. **b**: Seminal vesicle of animals treated with 10 pg kisspeptin. Number of secretary granules is lower. A mild dilatation of Golgi complex and endoplasmic reticulum is evident. ×5,000. **c**: Seminal vesicle of animals treated with 1 ng kisspeptin. Number of secretory granules was lower. Endoplasmic reticulum and Golgi apparatus were disorganized and dilated. Large vacuoles were evident. ×12,000. **d**: Seminal vesicle treated with 1 μg kisspeptin. Degeneration was more severe as compared to the other two treated groups. The alterations noticed were irregularity of nuclear shapes with intensive invagination of the envelope. No secretory granules were evident. ×15,000.

The second type of cells present in the secretory epithelium are basal cells. Basal cells were polygonal with their surfaces separated from the lumen by the apexes of the principal cells. They were occasionally present especially between two principal cells. They had compact cytoplasm. The cytoplasm contained few organelles, less well-developed Golgi apparatus and rough endoplasmic reticulum, and no inclusions other than lipid droplets. Their nuclei contained no nucleoli (Figure 3a).

At 10 pg kisspeptin dose transmission electron microscopy revealed significant changes in the secretory epithelium of seminal vesicles. These included dilatation in rough endoplasmic reticulum and Golgi apparatus dimensions, decrease in secretory granules and cell diameters. Degenerating as well as normal secretory granules were evident (Figure 3b).

At 1 ng kisspeptin dose the nuclei were swollen, hollow spaces and vacuoles were evident in the cytoplasm. Cytoplasm became compact. Number of secretory granules was lower. Endoplasmic reticulum was dilated (Figure 3c).

Degeneration was more severe at 1 μg kisspeptin dose as compared to the other two treated groups. The primary alterations noticeable were irregularity of the nuclear shapes with intensive invagination of the envelope, small nucleoli and prominent chromatin in the nuclear peripheral portion surrounding the nucleoli. The tissue became heterochromatic indicating low activity. Also noticeable were the granular endoplasmic reticulum and the Golgi complex with dilatation of the cisternae characterizing cellular atrophy and tissue disorganization of the organelles responsible for the secretory process. No secretory granules were evident. There was a significant reduction in cytoplasm (Figure 3d).

### DNA fragmentation

**Figure 4:**
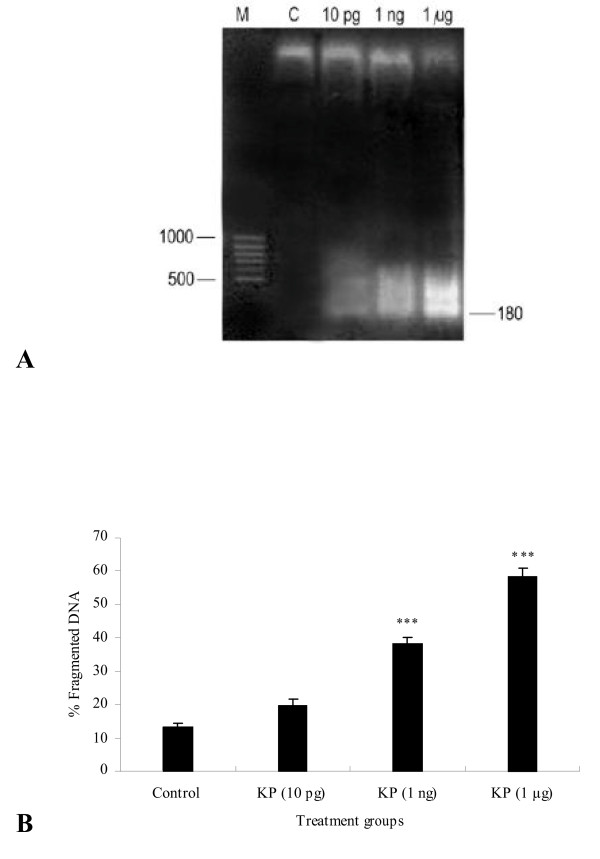
**DNA damage to control and kisspeptin treated seminal vesicles. A)** DNA ladder assay showing 180 bp bands of fragmented DNA, at 1 ng and 1 μg kisspeptin doses, from rat seminal vesicles. Faint bands were also visible in the 10 pg dose. Control (lane C) shows intact DNA. M = 100-bp DNA ladder. **B) **DNA fragmentation assay. Percentage of fragmented DNA relative to total DNA increased dose dependently with kisspeptin treatment. Values are expressed as mean ± SE. *P < 0.001.

In control rats, DNA fragmentation was scarcely visible in the seminal vesicle, while kisspeptin treatment at 10 pg, 1 ng and 1 μg doses 180-bp subunits were detected. The band intensity of fragmented DNA was highest in the 1 μg treated group but lower in 1 ng and 10 pg groups (Figure 4A).

Percent DNA fragmention gradually increased in rats administered higher kisspeptin doses. The % DNA fragmention increased in 10 pg treatment group but did not reach significant levels. However, it increased significantly (*P *< 0.001) in 1 ng and 1 μg groups as compared to control (Figure 4B).

## Discussion

Kisspeptin regulates the reproductive axis and acts as a molecular switch for onset of puberty. Over the last few years the role of kisspeptin in mammalian reproduction has been extensively studied. The peptide is secreted mainly by hypothalamic neurons and acting via stimulation of their cognate receptor GPR-54 on GnRH neurons, it regulates the secretion of pituitary gonadotropins to control steroid production at particular time and rate to initiate and maintain sexual maturity, while alteration in kisspeptin production may lead to abnormal gonadal function and impaired reproduction [34].

We hypothesize that kisspeptin induces early maturation of seminal vesicles. To this end, we administered kisspeptin-10 (i.p.) at three different doses twice daily for 12 days to 35 day old male prepubertal rats. Quite opposite to our hypothesis, the results demonstrated a dose dependent degeneration of seminal vesicles in prepubertal rats after kisspeptin administration.

The results of the present study demonstrate that continuous 12 days i.p. kisspeptin administration led to a dose dependent decrease in seminal vesicles weight and epithelial height of the secretory acini of seminal vesicle. In addition, cellular architecture was significantly modified and DNA fragmentation increased significantly at higher doses.

This is the first study to disclose the effects of kisspeptin on accessory sex glands in prepubertal rats. The adverse effects of kisspeptin on testicular tissue has been reported in prepubertal [35] and adult rats [23,36]. Thompson et al. (2006) reported that continuous administration of 50 nmol kisspeptin-54 for 13 days causes testicular degeneration in adult rats. Since Leydig cells are major source of testosterone production in testis, it is possible that kisspeptin treatment might have modulated Leydig cell function to reduce steroid production or promoted Leydig cell degeneration in treated animals. This might have contributed towards reduced plasma testosterone level in kisspeptin treated male rats. This possibility is further strengthened by the observations that kisspeptin significantly lowered plasma testosterone levels and thereby spermatogenesis in prepubertal rats [35]. Testosterone is essential for the maintenance of height of the mucosal epithelium required for continuous secretion of seminal vesicle. It also influences the function of smooth muscle in the seminal vesicles [37]. The growth and active secretion of seminal vesicle epithelium and other accessory reproductive tissues are dependent on the presence of circulating androgens [26,38]. Deprivation of androgens to seminal vesicles decreases cell size, structural integrity and morphological characteristics [38,39].

In the present study the Golgi complex and endoplasmic reticulum were dilated, number of secretory granules was lowered and characteristic structure of seminal vesicle was lost following kisspeptin administration. The dilatation of endoplasmic reticulum and Golgi complex may reflect a decrease in protein synthesis as previously evidenced by Veneziale et al. [40]. Secretory activity by the seminal vesicles is a sensitive, androgen dependent function. Decreased circulating testosterone levels in prepubertal rats [35] might ultimately led to atrophic changes.

Aumuller et al. [41] described that the formation of autophagic vacuoles and dilatation of cisternae of the Golgi complex and of the granular endoplasmic reticulum can be interpreted as signs of degradation of biological membranes.

Our results are in striking contrast to effects of kisspeptin in immature female rats [12] and also to those of Roa et al. (2008) [42] in terms of hormone concentrations, who reported significantly elevated serum FSH and LH levels at day 7 after constant i.c.v infusion of kisspeptin-10 to prepubertal female rats without effecting the uterine weight and the age of occurrence of vaginal opening. Redmond et al. (2011) [43] suggested a role for kisspeptin in the activation of the hypothalamic adenohypophyseal axis leading to the onset of puberty in ewe lambs. Although dose, duration and species differ, there exists some evidence in adult male rats [23], adult monkeys [21] adult and agonadal juvenile monkeys [22] and prepubertal male rats [35] that continuous administration of kisspeptin desensitizes kiss1r which eventually results in decreased LH secretion and testicular degeneration, while in females irrespective of dose, duration and species the results are completely different compared to males, leading to an early maturation of reproductive axis. However, it is not clear whether this finding reflects a difference in the way female respond to continuous exposure to kisspeptin compared with males or is due to differences between studies in the dose and mode of kisspeptin administration. Also Rhie et al. (2011) documented significantly elevated serum kisspeptin levels of Korean girls with central precocious puberty (CPP) compared to age-matched healthy prepubertal controls. CPP in females is therefore, likely to be triggered by premature increase of kisspeptin [44].

## Conclusions

The results obtained in this study imply that DNA damage, histological and ultrastructural changes in seminal vesicles are suggestive of degeneration of seminal vesicles after intraperitoneal twice daily administration of kisspeptin.

## Competing interests

The authors declare that they have no competing interests.

## Authors' contributions

FR has conducted the entire work, designed the study and drafted the manuscript. IZQ has supervised the study. MR and MHR has contributed the data analysis. FR has contributed to histology. All authors read and authorized the final manuscript.
